# Genetic engineering of a temperate phage-based delivery system for CRISPR/Cas9 antimicrobials against *Staphylococcus aureus*

**DOI:** 10.1038/srep44929

**Published:** 2017-03-21

**Authors:** Joo Youn Park, Bo Youn Moon, Juw Won Park, Justin A. Thornton, Yong Ho Park, Keun Seok Seo

**Affiliations:** 1 Department of Basic Sciences, College of Veterinary Medicine, Mississippi State University, Mississippi State, MS 39762, United States; 2 Department of Microbiology, College of Veterinary Medicine and BK21 Program for Veterinary Science, Seoul National University, Seoul 151-742, South Korea; 3 Department of Computer Engineering and Computer Science, KBRIN, University of Louisville, KY 40208, United States; 4 Department of Biological Sciences, Mississippi State University, Mississippi State, MS 39762, United States

## Abstract

Discovery of clustered, regularly interspaced, short palindromic repeats and the Cas9 RNA-guided nuclease (CRISPR/Cas9) system provides a new opportunity to create programmable gene-specific antimicrobials that are far less likely to drive resistance than conventional antibiotics. However, the practical therapeutic use of CRISPR/Cas9 is still questionable due to current shortcomings in phage-based delivery systems such as inefficient delivery, narrow host range, and potential transfer of virulence genes by generalized transduction. In this study, we demonstrate genetic engineering strategies to overcome these shortcomings by integrating CRISPR/Cas9 system into a temperate phage genome, removing major virulence genes from the host chromosome, and expanding host specificity of the phage by complementing tail fiber protein. This significantly improved the efficacy and safety of CRISPR/Cas9 antimicrobials to therapeutic levels in both *in vitro* and *in vivo* assays. The genetic engineering tools and resources established in this study are expected to provide an efficacious and safe CRISPR/Cas9 antimicrobial, broadly applicable to *Staphylococcus aureus*.

Invasive infections with methicillin resistant *Staphylococcus aureus* (MRSA) in both community and healthcare settings total approximately 80,000/year and accounted for 11,285 deaths in 2011, resulting in direct heath care costs of more than $4.5 billion in the United States alone^1^,^2^. Moreover, the increasing occurrence of vancomycin-intermediate *S. aureus* (reduced efficacy of vancomycin) resulted from an accumulation of single nucleotide polymorphisms in the *S. aureus* chromosome by long-term exposure to vancomycin^3^,^4^,^5^. The increasing frequency of this problem underlines an urgent need for new antibiotics. However, the numbers of newly developed antibiotics and commercial interest in such drugs are decreasing, due to the high costs in development and rapidly rising resistance^6^. These impediments have led to an interest in the development of alternative therapeutics such as vaccines, probiotics, and phage therapy that are less likely to drive resistance.

The CRISPR (Clustered, regularly interspaced, short palindromic repeats) and CRISPR associated (Cas) genes serve as a bacterial immune system to resist foreign DNA^7^,^8^. The Cas9 present in the Type II CRISPR/Cas system of *Streptococcus pyogenes* is a RNA-guided endonuclease that introduces double-stranded breaks into target genes^9^. The specificity of Cas9 is guided by a trans-activating small RNA (tracrRNA) and CRISPR RNA (crRNA) harboring a short spacer sequence recognizing the target gene^10^,^11^. Recent studies demonstrated that a plasmid or phagemid harboring a CRISPR/Cas9 system programmed to target an antibiotic resistance gene or a specific pathogen could be delivered by a temperate phage and could successfully control antibiotic resistant *Escherichia coli* or MRSA with minimal effects on non-targeted bacteria^12^,^13^,^14^,^15^,^16^. These studies demonstrated the potential use of CRISPR/Cas9 system as a programmable antimicrobial to selectively control the target bacteria at the DNA level without disturbing the normal microbiome^12^,^13^,^14^,^15^,^16^. However, the efficacy of CRISPR/Cas9 antimicrobials is still far from being therapeutic, mainly due to the low efficiency in phage-based delivery systems which limited the efficacy of CRISPR/Cas9 to reduce bacterial colony forming units (CFU) by only one or two logs in *in vivo* and *in vitro* assays^12^,^14^. Furthermore, phage-based delivery systems may deliver not only a plasmid or phagemid harboring CRISPR/Cas system, but also host chromosomal segments by generalized and specialized transduction to target cells^17^. This is particularly important for phage-based delivery systems using *S. aureus* since many important staphylococcal virulence factors such as superantigens and cytolysins are commonly located in mobile genetic elements (MGEs) and are transferred to other *S. aureus* and *Listeria monocytogenes* by temperate phage-mediated generalized transduction^18^,^19^, thereby raising the safety issues^20^,^21^,^22^.

In this study, we demonstrate a genetic engineering strategy to overcome shortcomings in phage-based delivery systems by integrating the CRISPR/Cas9 system into the genome of temperate phage. The modifications allow for improved efficiency of delivery to target cells, expanded host specificity by complementing the tail fiber protein of the phage, and removal of virulence factor genes from the host strain to prevent contamination of harmful bacterial products in the phage lysates and subsequent spread of virulence genes by generalized transduction.

## Results

### Development of programmable and integrative CRISPR/Cas9 plasmid vector systems

**Figure 1:**
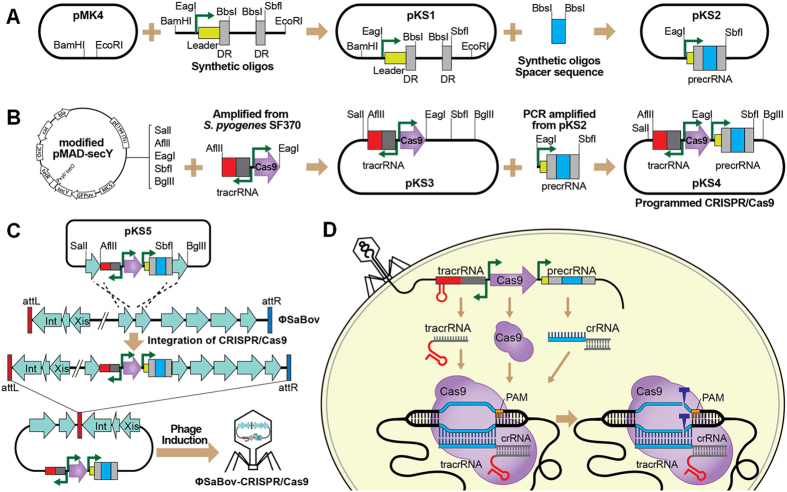
A schematic map of programmable and integrative CRISPR/Cas9 system. (**A**) To generate a programmable CRISPR/Cas9 system, synthetic oligos containing a promoter, leader sequence and direct repeat (DR) sequence flanked with the *Bbs*I restriction sites (Supplemental Figure S1) were cloned into the pMK4 shuttle vector, resulting pKS1. Synthetic oligos specific to the target gene (spacer sequence) were cloned into the *Bbs*I site, resulting pKS2. (**B**) To generate integrative CRISPR/Cas9 system, genes encoding tracr-RNA and Cas9 were amplified from chromosomal DNA of *Streptococcus pyogenes* SF370 using PCR and cloned into the modified pMAD-secY temperature sensitive shuttle vector, resulting pKS3. To program CRISPR/Cas9 system specific to the target gene, the pre-crRNA (the promoter, leader sequence, DR, and spacer sequence) was amplified from pKS2 and cloned into pKS3, resulting pKS4. (**C**) The programmed CRISPR/Cas9 system was integrated into the non-coding region of ϕSaBov genome by homologous recombinations. (**D**) The CRISPR/Cas9 system programmed to target S. aureus is induced and delivered by ϕSaBov. The CRISPR/Cas9 system (tracrRNA, crRNA, and Cas9) is expressed and scanned the PAM sequence and recognize the target sequence in the chromosomal DNA, leading to chromosomal DNA cleavage and bacterial death.

The fact that bacteriophages can package their own genome more efficiently than host genetic elements, such as plasmids and phagemids, inspired us to develop a programmable and integrative vector system containing CRISPR/Cas9 integrated within the phage genome. This strategy was designed to improve packaging and delivery of the CRISPR system to target cells. To generate a programmable CRISPR/Cas system, synthetic oligonucleotides containing a CRISPR array encoding a promoter, leader sequence, and direct repeats interspaced with two *Bbs*I restriction sites (Supplemental Figure S1) was cloned into pMK4, resulting pKS1 (Fig. 1A). Synthetic oligos containing a spacer sequence specific to the *nuc* gene, uniquely present in all *S. aureus*, followed by a protospacer adjacent motif (PAM) NGG, was cloned into *Bbs*I sites in pKS1, resulting in pKS2 (Fig. 1A). To generate integrative CRISPR/Cas9 system, tracrRNA and Cas9 genes were amplified from CRISPR/Cas9 system of *Streptococcus pyogenes* and cloned into the modified pMAD-secY temperature sensitive shuttle vector system developed in this study (Supplemental Figure S2), resulting in pKS3 (Fig. 1B). To program CRISPR/Cas9 system specific to *S. aureus*, CRISPR array with a spacer sequence specific to the *nuc* gene cloned in pKS2 was amplified by PCR and cloned into pKS3, resulting pKS4 (Fig. 1B). This programmed CRISPR/Cas9 system was integrated into the genome by allelic exchange as described below.

### Integration of CRISPR/Cas9 system into the genome of ϕSaBov lysogenized in *S. aureus* strain RF122

**Figure 2:**
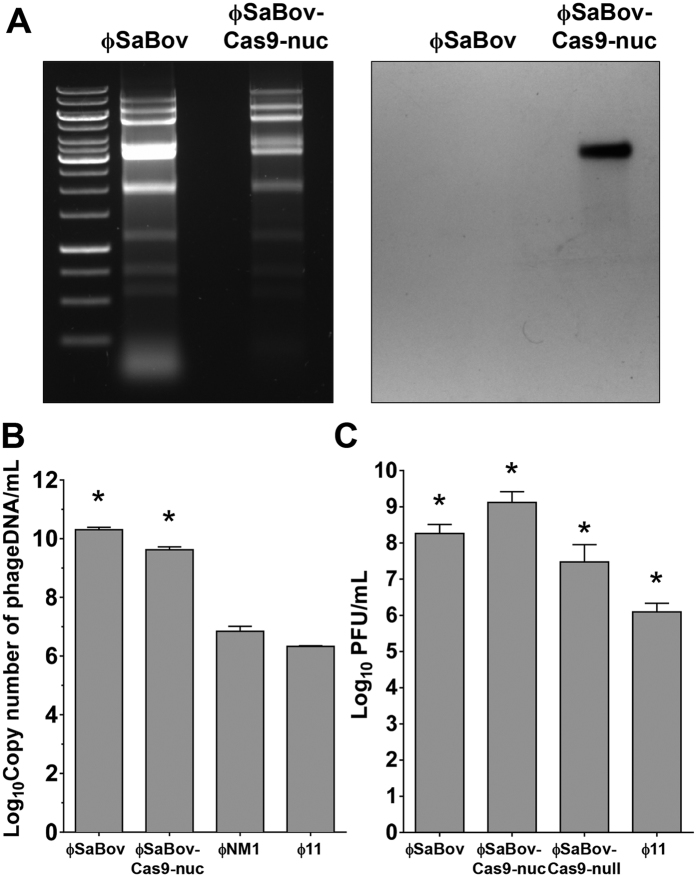
Characterizing the ϕSaBov integrated with CRISPR/Cas9 system. (**A**) Integration of CRISPR/Cas9 system specific to *S. aureus* was verified by southern blot analysis using a probe specific to the leader sequence. (**B**) The copy number of phage DNA in the phage lysates was determined using quantitative real time PCR and standard curves presented in Supplemental Figure 3. (**C**) The number of transducing phage particles in the phage lysates was determined by calculating the plaque forming unit (PFU) using semi-solid agar overlay method. The bar graph indicates the average and SEM combined from triple measurements of three independent experiments (n = 9). Asterisk indicates statistical significance in student *t*-test, compared to the results from ϕ11 (p < 0.001).

In order to select staphylococcal temperate phages efficiently packaging their own phage genomes, we determined the absolute copy number of phage DNA in the phage lysates using quantitative real time PCR (qRT-PCR) and standard curves generated from serially diluted plasmid templates (Supplemental Figure S3). Since a single transducing phage particle harbors a single copy of phage DNA, the copy number of phage DNA is equal to the number of transducing phage particles. We found that the ϕSaBov lysogenized in *S. aureus* strain RF122, a bovine mastitis isolate belonging to CC151 lineage, generated an exceptionally high number of transducing phage particles (10.31 Log copies/ml phage lysate) which was 3.44 and 3.98 Log magnitude higher than that of ϕ11 and ϕNM1, respectively (Fig. 2A). Based on these results, we selected the ϕSaBov for phage-based CRISPR/Cas9 delivery system.

To integrate the *S. aureus*-specific, programmed CRISPR/Cas9 system from pKS4 into the genome of ϕSaBov, upstream and downstream gene segments of non-coding regions between SAB1737 and SAB1738 of the ϕSaBov genome were amplified by PCR and cloned into pKS4, resulting pKS5 (Fig. 1C). This plasmid was transformed into the strain RF122Δ*nuc* ϕSaBov in which the *nuc* gene has been removed to prevent CRISPR/Cas9 mediated-killing. The programmed CRISPR/Cas9 system was integrated into the genome of ϕSaBov by allelic exchange, resulting in RF122Δ*nuc* ϕSaBov-Cas9-nuc (Fig. 1C). Phages induced from this strain were referred as to ϕSaBov-Cas9-nuc. Southern blot analysis using a probe specific to the pre-crRNA gene confirmed the integration of CRISPR/Cas system into the genome of ϕSaBov (Fig. 2A). The copy number of recombinant phage genome harboring CRISPR/Cas9 system (ϕSaBov-Cas9-nuc) was determined by quantitative real time PCR (qRT-PCR) and showed a slight decrease, compared to those of wild type ϕSaBov, possibly due to the increase of genome size following integration of CRISPR/Cas9 system, but was still significantly higher than ϕ11 and ϕNM1 (Fig. 2B). The transduction efficacy of the ϕSaBov-Cas9-nuc was determined by calculating the plaque forming unit (PFU) and showed that the PFU of ϕSaBov-Cas9-nuc was higher than that of wild type ϕSaBov, presumably due to the killing effect of CRISPR/Cas9 system in ϕSaBov-Cas9-nuc which prevents lysogenic conversion events by ϕSaBov (Fig. 2C).

As a control, CRISPR/Cas9 system without a spacer sequence was integrated into the genome of ϕSaBov in RF122Δ*nuc*. Phages induced from this strain were referred as to ϕSaBov-Cas9-null.

### The specificity and efficacy of ϕSaBov-Cas9-nuc in *in vitro* assays

**Figure 3:**
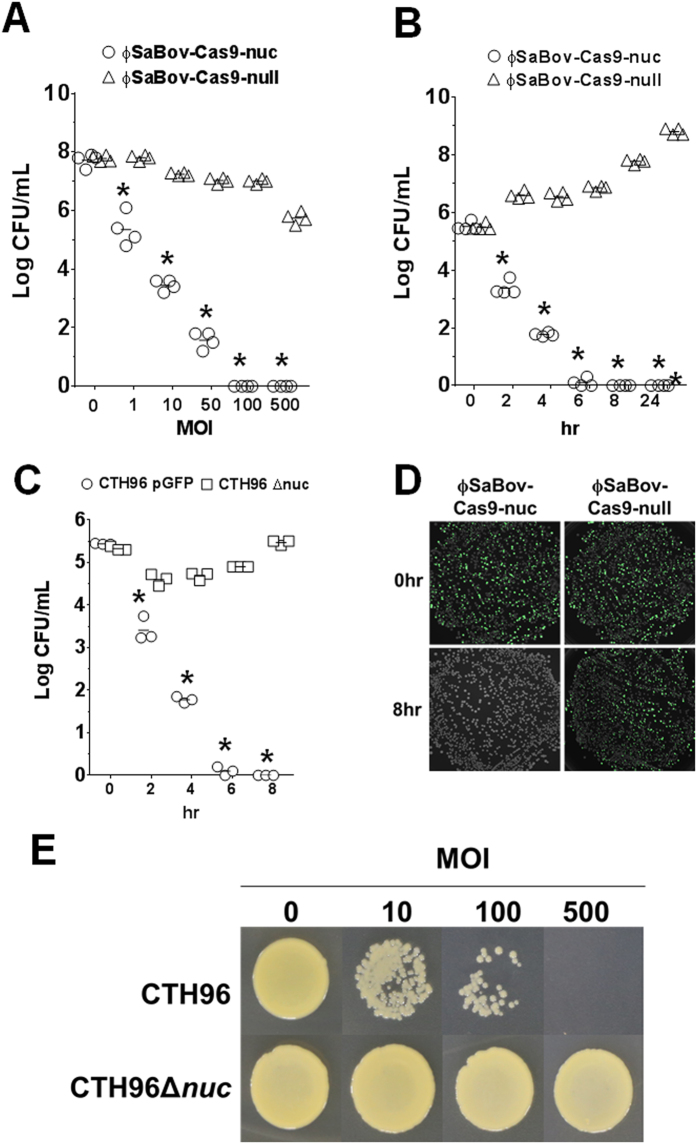
The efficacy and specificity of ϕSaBov-Cas9-nuc in *in vitro* assays. A mid exponential culture of *S. aureus* strain CTH96 (1 × 10^5^ CFU) was treated (**A**) at various MOIs of ϕSaBov-Cas9-nuc or ϕSaBov-Cas9-null for 6 h or (**B**) at 50 MOI of ϕSaBov-Cas9-nuc or ϕSaBov-Cas9-null up to 24 h. Viable cells were recovered on BHI plates. Data point represent the average of triple measurements which were repeated in four independent experiments. (**C**) A mid exponential culture of *S. aureus* strain CTH96pGFP or CTH96Δ*nuc* (1 × 10^5^ CFU) was treated at 50 MOI of ϕSaBov-Cas9-nuc for 8 h. Viable cells were recovered on BHI plates. Data points indicate the average of triple measurements which were repeated in three independent experiments. Asterisk indicates statistical significance in student *t*-test, compared to the results from ϕSaBov-Cas9-null (p < 0.001). (**D**) A mixture of *S. aureus* strain CTH96pGFP and CTH96Δ*nuc* (1:1, each at 5 × 10^4^ CFU) was treated with ϕSaBov-Cas9-nuc at MOI of 50 for 8 h. Viable cells were recovered from BHI plates. Pictures showing expression of green fluorescent protein were obtained under UV wave length using Gel Doc system (Bio-Rad). Results shown are a representative picture repeated in three independent experiments. (**E**) A sterile empty antibiotic disc was inoculated with a suspension of CTH96 or CTH96Δ*int* and treated with the ϕSaBov-Cas9-nuc at various MOIs for 8 h. Viable cells were recovered by blotting discs onto BHI plates. Results shown are a representative picture repeated in three independent experiments.

To assess the efficacy of killing by ϕSaBov-Cas9-nuc, *S. aureus* strain CTH96, a bovine isolate susceptible to ϕSaBov, was treated with various multiplicities of infection (MOIs) of ϕSaBov-Cas9-nuc, and viable cells were recovered by plating on BHI agar. We defined the MOI as the number of transducing phage particles per recipient cell. When treated for 6 h, recipient cells were completely killed at MOI of 100 or above, and 5.01% and 0.08% of viable recipient cells were recovered at MOI of 10 and 50, respectively (Fig. 3A). In order to assess the natural occurrence of survival due to the spontaneous mutations in targets, a time-dependent killing was measured. Upon treatment with ϕSaBov-Cas9-nuc at MOI of 50, the number of viable recipient cells gradually decreased, and no viable cells were recovered after 8 h treatment and sustained at 24 h, suggesting rare occurrence of surviving mutants (Fig. 3B). By contrast, treatment with ϕSaBov-Cas9-null did not show any killing effect at MOI of 50 or less and minor killing effects at MOI of 100 or above, presumably due to the lytic cycle of ϕSaBov (Fig. 3A and B).

To assess the *nuc* gene specific killing effect of ϕSaBov-Cas9-nuc, we generated the *nuc* gene deletion mutant of CTH96 (CTH96Δ*nuc*) lacking the target gene for a spacer sequence in CRISPR/Cas9 system and CTH96 carrying a plasmid expressing green fluorescent protein (CTH96pGFPuv). When treated with ϕSaBov-Cas9-nuc at a MOI of 50, viable CTH96pGFPuv gradually decreased and completely lost viability at 8 h of treatment (Fig. 3C). By contrast, the number of viable CTH96Δ*nuc* slightly decreased within 2 h of treatment, presumably due to the lytic cycle of ϕSaBov, and then gradually increased thereafter (Fig. 3C). Next, the mixed cultures of CTH96Δ*nuc* and CTH96pGFPuv were treated with ϕSaBov-Cas9-nuc or ϕSaBov-Cas9-null at MOI of 50 for 8 h, and viable cells recovered on BHI plates were analyzed under the UV lamp. When treated with the ϕSaBov-Cas9-nuc, recipient cells expressing GFP were selectively killed (Fig. 3D). By contrast, when treated with the ϕSaBov-Cas9-null, both CTH96Δ*nuc* and CTH96pGFPuv were equally recovered (Fig. 3D). The killing effect of ϕSaBov-Cas9-nuc against *S. intermedius* (coagulase positive staphylococci) and *S. epidermidis* (coagulase negative staphylococci) was also tested but was not observed (data not shown). Combined, these results clearly demonstrated the *nuc* gene-specific killing effect of ϕSaBov-Cas9-nuc.

The effect of CRISPR/Cas9 system requires biological activities including transcription and translation of the CRISPR/Cas9 system within the recipient cells which might limit the application of CRISPR/Cas9 antimicrobials under the nutritionally and metabolically-limited conditions. To simulate the nutritionally and metabolically-limited conditions, the empty antibiotic disc was inoculated with *S. aureus* in PBS (1 × 10^5^ CFU), followed by treatment with the ϕSaBov-Cas9-nuc at MOIs of 10, 100, and 500. After 8 h treatment, viable cells were recovered by blotting the disc on to the BHI plate. Recipient cells were gradually decreased at MOIs of 10 and 100, and completely decolonized at MOIs of 500 (Fig. 3E). By contrast, the viability of recipient cells lacking the *nuc* gene (CTH96Δ*nuc*) was not affected (Fig. 3E). These results suggest the potential use of ϕSaBov-Cas9-nuc for sanitizing abiotic objects such as medical devices or surfaces.

We chose the *nuc* gene as a target for CRISPR/Cas9 system because the *nuc* gene is uniquely present in all *S. aureus*, thus CRISRR/Cas9 system only kills *S. aureus* without affecting microbiomes. To determine whether the efficacy of CRISPR/Cas9 killing effect is affected by the spacer gene, we generated the CRISPR/Cas9 system targeting the *esxA* (ϕSaBov-Cas9-esxA) which is also highly conserved in all sequenced *S. aureus* strains. The efficacy of ϕSaBov-Cas9-esxA was not significantly different from that of ϕSaBov-Cas9-nuc (Supplemental Figure S4). We tested the efficacy of CRISPR/Cas9 system targeting two proximate locations of the *nuc*, thereby, cleaving two chromosomal locations within the *nuc* gene which also showed a similar killing effect to CRISPR/Cas9 targeting a single location (data not shown). These results suggest that the efficacy of CRISPR/Cas9 killing effect is mainly determined by the efficiency of CRISPR/Cas9 system delivery to the target organisms.

### The efficacy of ϕSaBov-Cas9-nuc in *in vivo* assays

**Figure 4:**
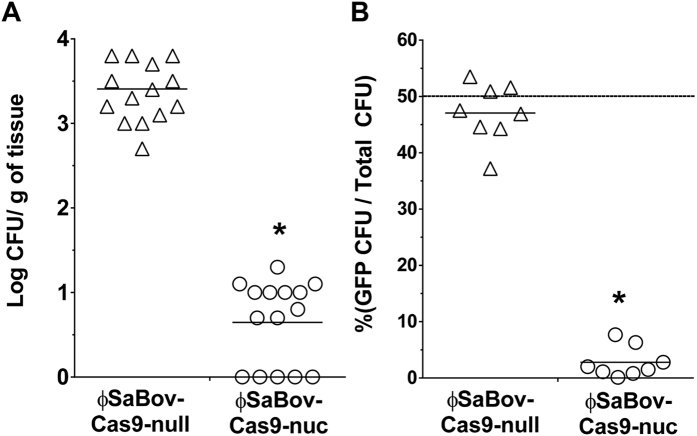
The efficacy of ϕSaBov-Cas9-nuc in *in vivo* murine skin infection. The backs of C57BL/6 mice were intradermally infected with a suspension of (**A**) CTH96pGFP (1 × 10^5^ CFU) or (**B**) a mixture of CTH96pGFP and CTH96Δ*nuc* (1:1, each at 5 × 10^4^ CFU) for 6 h, followed by treatment with the ϕSaBov-Cas9-nuc or ϕSaBov-Cas9-null at MOI of 500. After 24 h, infected skin was excised and homogenized. Viable cells were recovered by plating serially diluted homogenates onto BHI plate. The specificity of ϕSaBov-Cas9-nuc was evaluated by the proportion of viable cells expressing green fluorescent protein in total viable cells. Data points indicate the average of triple measurements in individual mice (n = 9). Asterisk indicates statistical significance in student *t*-test, compared to the results from ϕSaBov-Cas9-null (p < 0.001).

To test the efficacy of ϕSaBov-Cas9-nuc *in vivo*, the backs of C57BL/6 mice were shaved and intradermally inoculated with recipient cells (CTH96pGFP, 1 × 10^5^ CFU). After 6 h of infection, ϕSaBov-Cas9-nuc or ϕSaBov-Cas9-null was injected into the infected skin at an MOI of 500. Following treatment for 24 h, infected skin regions were excised and homogenized to recover viable cells. The number of viable cells recovered from the skins treated with ϕSaBov-Cas9-nuc (0.647 ± 0.128 Log CFU/g of tissue, mean ± SEM) was significantly lower than that treated with ϕSaBov-Cas9-null (3.333 ± 0.131 Log CFU/g of tissue) (Fig. 4A). Next, to test the *nuc* gene-specific killing capacity of ϕSaBov-Cas9-nuc *in vivo*, animals were infected with a mixture of CTH96pGFP and CTH96Δ*nuc* (1:1, each at 5 × 10^4^ CFU) for 6 h, followed by treatment with ϕSaBov-Cas9-nuc or ϕSaBov-Cas9-null at MOI of 500 for 24 h. The proportion of viable cells expressing GFP in infected skin treated with ϕSaBov-Cas9-nuc was 2.8 ± 2.6%, in contrast to those treated with ϕSaBov-Cas9-null showing 47.1 ± 4.9% (Fig. 4B), suggesting *nuc* gene-specific killing effect by ϕSaBov-Cas9-nuc *in vivo*.

**Figure 5:**
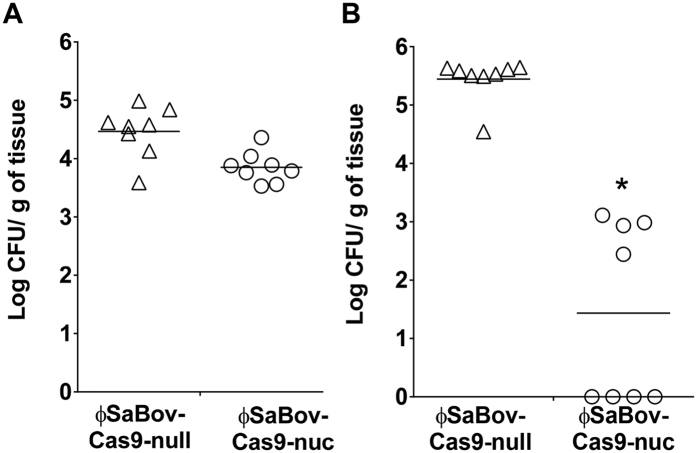
The efficacy of ϕSaBov-cas9-nuc in decolonization of *S. aureus* from surface of skin. The back skin of mice was shaved, depilated, and decolonized with 70% alcohol, followed by recolonization with CTH96pGFP (2 × 10^4^ CFU). After 6 h, ϕSaBov-Cas9-nuc or ϕSaBov-Cas9-null at MOI of 500 was topically applied in solution (**A**) or in mixing with a hydrogel (**B**). Following treatment for 24 h, infected skins were dissected and homogenized to determine the viable cell count. Data points indicate the average of triple measurements in individual mice (n = 8). Asterisk indicates statistical significance in student *t*-test, compared to the results from ϕSaBov-Cas9-null (p < 0.001).

Lastly, we tested if the ϕSaBov-Cas9-nuc was able to decolonize *S. aureus* from the surface of skin. The back of mice skin were shaved, depilated, decolonized with 70% alcohol, and colonized with CTH96pGFP (2 × 10^4^ CFU) by cotton swab. After 6 h, ϕSaBov-Cas9-nuc or ϕSaBov-Cas9-null at MOI of 500 was topically applied by spraying. Following treatment for 24 h, infected skins were dissected and homogenized to determine the viable cell count. However, the number of viable cells recovered from infected skins treated with ϕSaBov-Cas9-nuc was not significantly different from that treated with ϕSaBov-Cas9-null (Fig. 5A). We noticed that the surface of skin was completely dried in 15 mins from the application of inoculum, or phage solution that may create an environment with limited water activity which suppress transcriptional and translational activities of *S. aureus*, thereby the machinery of CRISPR/Cas antimicrobials could not be expressed. In order to maintain water activity, ϕSaBov-Cas9-nuc or ϕSaBov-Cas9-null was mixed with a hydrogel and topically applied to the infected skin by swabbing which significantly improved the efficacy of killing effect of ϕSaBov-Cas9-nuc (Fig. 5B).

### Prevention of toxins contaminations in phage lysates

**Figure 6:**
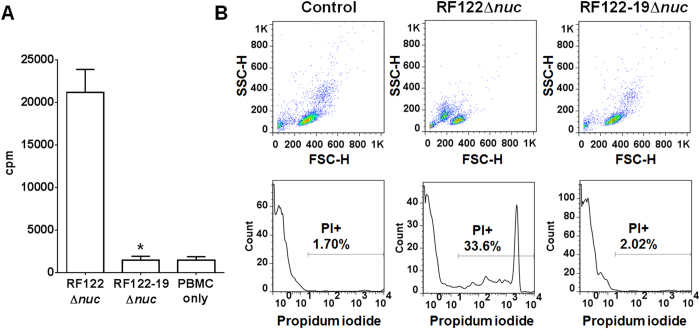
Prevention of toxin contamination in phage lysates. Human PBMCs were treated with phage lysates generated from RF122Δ*nuc* or RF122-19Δ*nuc*, an isogenic strain lacking 10 superantigen and 11 cytotoxin genes. (**A**) After 3 day incubation, proliferation of T cells caused by superantigens in phage lysages was measured by incorporation of radioactive ^3^H-thymidine into the cellular DNA using liquid scintillation counter. Bars indicate the count per minute (cpm) of radioactivity combined from triple measurements of three independent experiments (n = 9). Asterisk indicates statistical significance in student *t*-test, compared to the results from the RF122Δ*nuc* (p < 0.001). (**B**) After 3 h incubation, cytotoxicity caused by cytotoxins in phage lysates was measured by incorporation of propidium iodide into dead cells (propidium iodide positive, PI+) using flow cytometry. Data shown are representative results repeated in three independent experiments.

The strain RF122 harbors 10 superantigens (*sec, seg, sei, selm, seln, selo, selu, sell, tst1, selx*) and 11 cytolysins (*hla, hlb, hlgA, hlgB, hlgC, lukD, lukE, lukG, lukH, lukM, lukF’*) genes (Supplemental Figure S5) which could be contaminated in the phage lysates generated from the strain RF122. Furthermore, our previous studies demonstrated that induction of ϕSaBov from strain RF122 generated transducing phage particles harboring genetic segments specifically associated with MGEs such as νSaα, νSaβ, νSaγ, and SaPI1 some of which contain superantigens (*sec, seg, sei, selm, seln, selo, selu, tst*) and cytolysins (*hla, lukD, lukE*) and transferred these virulence genes to other *S. aureus* strains by generalized transduction^23^,^24^. To prevent contamination of these toxins in phage lysates and potential spread of toxin genes by the ϕSaBov-based delivery system, 10 superantigens and 11 cytotoxins present in the chromosome of RF122Δ*nuc* ϕSaBov-Cas9-nuc were removed by allelic exchange using a modified pMAD-secY system, resulting in RF122-19Δ*nuc* ϕSaBov-Cas9-nuc. The deletions were confirmed by PCR analysis (Supplemental Figure S5). As expected, phage lysates generated from the strain RF122Δ*nuc* showed superantigenicity as indicated by significantly higher count per minute (cpm) as a result of incorporation of [^3^H]-thymidine into dividing cellular DNA (Fig. 6A). Consistently, incubation with phage lysates generated from the strain RF122Δ*nuc* induced cytotoxicity by cytolysins in phage lysates as demonstrated by intercalation of propidium iodide into cellular DNA (Fig. 6B). By contrast, phage lysates generated from the strain RF122-19Δ*nuc* did not induce any superantigenicity or cytotoxicity (Fig. 6A and B).

### Expansion of host specificity of ϕSaBov

**Figure 7:**
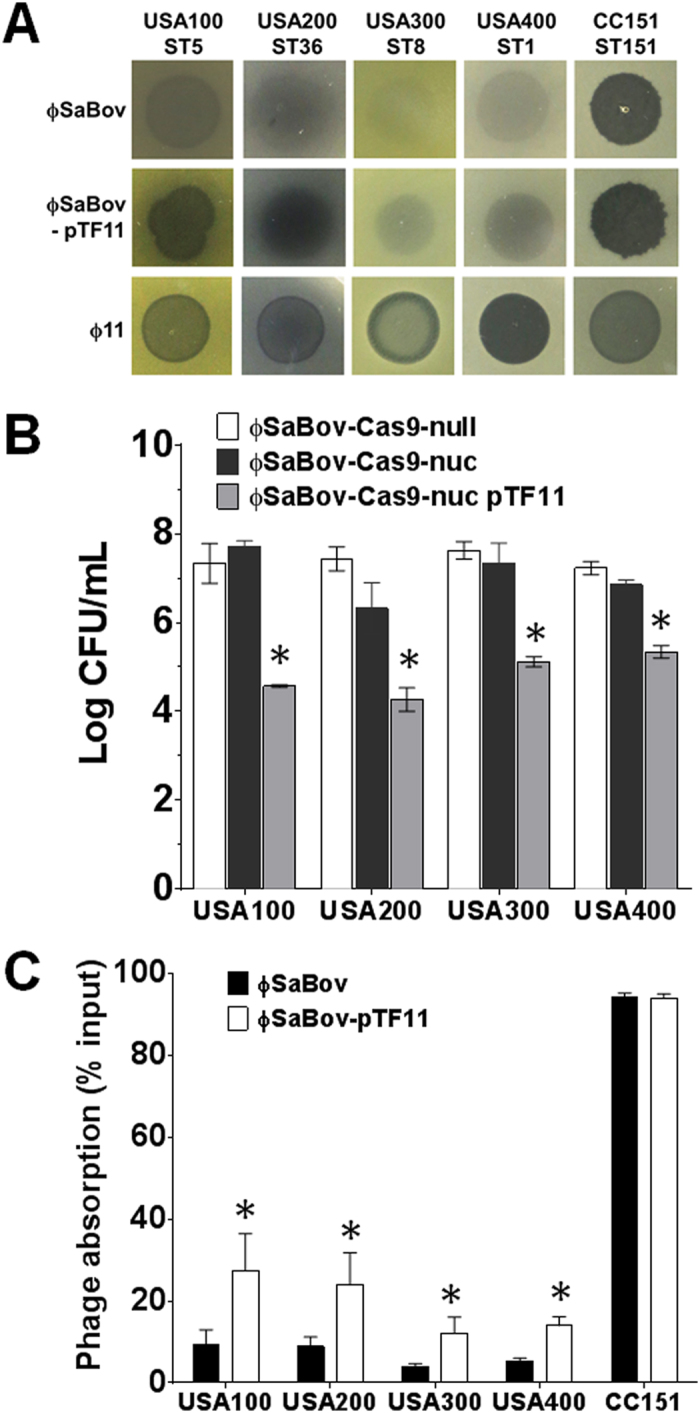
Expansion of host specificity of ϕSaBov by complementing the tail fiber protein. (**A**) For phage spot test, 10 μL of phage lysates (of ϕ11, ϕSaBov, or ϕSaBov complemented with the tail fiber protein of ϕ11 (ϕSaBov-pTF11) containing 1 × 10^8^ pfu/ml was inoculated onto the lawn culture of several pandemic human clones of *S. aureus*. (**B**) To measure the efficacy of CRISPR/Cas9 system delivered by ϕSaBov complemented with the tail fiber protein of ϕ11, a mid-exponential culture of pandemic human *S. aureus* clones (1 × 10^5^ CFU) was treated with phage lysates of ϕSaBov-Cas9-null, ϕSaBov-Cas9-nuc, or ϕSaBov-Cas9-nuc pTF11 at MOI of 100 for 8 h. The bar graph indicates the average and SEM of Log CFU of viable cells, combined from triple measurements of three independent experiments (n = 9). (**C**) The phage absorption was determined by calculating the proportion of unbound phage from the total phage input (2.5 × 10^5^ pfu) to several pandemic human clones of *S. aureus* (5 × 10^7^ CFU) at the MOI of 0.005. Asterisk indicates statistical significance in student *t*-test, compared to the results from ϕSaBov (p < 0.001).

The ϕSaBov has a narrow host range highly specific to bovine CC151 lineage of *S. aureus* as shown in phage spot test (Fig. 7A, ϕSaBov panel), thereby the efficacy of ϕSaBov-Cas9-nuc to human pandemic clonal lineage of *S. aureus* (ST1, ST5, ST8 and ST36) was minimal or had no effect (Fig. 7B, black bars). Host specificity of phage is primarily determined by phage-encoded tail fiber proteins interacting with receptors on host cells such as membrane proteins or cell wall carbohydrates^25^,^26^,^27^. We investigated the possible expansion of host ranges of ϕSaBov by complementing the gene encoding the tail fiber protein (Tif) from ϕ11 (orf50)^28^ which has a broad spectrum of host range in several pandemic clones of human *S. aureus* isolates in the United States (Fig. 7A, ϕ11 panel). A pMK4 shuttle vector harboring the tail fiber protein gene of ϕ11 (pTF11) was constructed and transformed into the strain RF122-19Δ*nuc* ϕSaBov and RF122-19Δ*nuc* ϕSaBov-Cas9-nuc, resulting RF122-19Δ*nuc* ϕSaBov-pTF11 and RF122-19Δ*nuc* ϕSaBov-Cas9-nuc-pTF11, respectively. Phages induced from RF122 Δ*nuc* ϕSaBov-pTF11 (ϕSaBov-pTF11) showed improved clear zone of lysis in phage spot tests against pandemic human clonal lineages (ST1, ST5, ST8 and ST36) of *S. aureus* strains (Fig. 7A, ϕSaBov-pTF11 panel), compared to that of ϕSaBov. Phage absorption assay showed that the complementation of the Tif from ϕ11 significantly improved the phage absorption to the pandemic human clonal lineage, albeit not as high as to the CC151 (Fig. 7C). Consistently, phages induced from RF122 Δ*nuc* ϕSaBov-Cas9-nuc pTF11 significantly improved the killing effect of CRISPR/Cas9 against ST1, ST5, ST8 and ST36 lineages of *S. aureus* ranging from 1.51 to 3.15 order of magnitude (Fig. 7B, gray bars).

## Discussion

Conventional antibiotics, targeting proteins of critical bacterial cellular pathways, are often rendered ineffective due to bacteria either acquiring episomes harboring resistance genes or accruing spontaneous mutations in targets^6^. The CRISPR/Cas9 antimicrobials have become an attractive alternative due to the advantages of sequence-specific killing without disturbing the microbiome and multiplex features of spacer sequences to simultaneous target multiple genes, thereby preventing development of resistant mutants^29^. Despite promising results, a therapeutic use of CRISPR/Cas9 antimicrobials is still far from being practical due to the shortcomings in efficiency of delivery and safety aspects of phage-based delivery systems^12^,^14^. In this study, we demonstrated a novel genetic engineering strategy to enhance the efficacy and safety of phage-based delivery systems by integrating CRISPR/Cas9 system into the genome of a temperate phage to improve the delivery to target cells, complementing phage tail fiber protein to extend the host spectrum, and removing virulence genes from the host strain to prevent contamination by toxins and spread of virulence genes.

We chose the ϕSaBov lysogenized in *S. aureus* strain RF122 as a candidate for phage-based CRISPR/Cas9 delivery system because induction of ϕSaBov from the strain RF122 generated an exceptionally high number of transducing phage particles harboring the phage genome. Indeed, integration of CRISPR/Cas9 system into the genome of ϕSaBov significantly enhanced the efficacy of *S. aureus* specific-killing by ϕSaBov-Cas9-nuc to nearly complete decolonization *in vitro* under both nutritionally enriched and limited conditions and more than two orders of magnitude CFU reduction in an *in vivo* murine skin infection experiments. Interestingly, efficient packaging of the ϕSaBov genome is highly specific to the RF122 background, and not reproduced in other strains such as RN4220 (Supplemental Figure 6) or MW2^23^. These results suggest the presence of genetic elements uniquely present in the chromosome of RF122 promoting phage DNA excision and replication. Phage DNA excision, replication, and packaging are controlled by complex mechanisms involving multiple factors encoded in the phage genome and host chromosome. Upon induction of phage by SOS signals, phage-encoded *rinA* and *rinB* activate transcription of phage-encoded integrase (Int), excisionase (Xis), and unknown host encoded factors such as IHF and Fis to initiate site-specific recombination at the attachment site (*att* site)^28^,^30^,^31^. Genome sequence comparison of RF122, MW2, and RN4220 revealed several unique integrases, transposases, and integrative and conjugative elements associated with MGEs and reminiscent of inactivated phage present in the chromosome of RF122^32^. We are currently investigating the role of these unique genetic elements present in RF122 on efficient phage DNA excision and packaging events by ϕSaBov.

Our results showed that the application conditions may greatly affect the efficacy of CRISPR/Cas9 mediated killing effect. When applied in the dry skin condition, the ϕSaBov-Cas9-nuc was unable to decolonize *S. aureus* from the skin surface, presumably due to the low water activity suppresses transcriptional and translational activities of *S. aureus* in which the machinery of CRISPR/Cas9 antimicrobial could not be expressed. By contrast, when mixed with a hydrogel to support water activity, the ϕSaBov-Cas9-nuc successfully decolonized *S. aureus* from the infected skin surface. It is also possible that intravenous administration of CRISPR/Cas antimicrobials delivered by phage lysates may evoke immune responses by transducing phage particles or bacterial products remaining in the phage lysates, resulting in antibody production, decreasing efficacy, and potential allergic reactions. Therefore, the most practical application of CRISPR/Cas antimicrobials delivered by phage lysates would be topical application to the condition supporting water activity such as infected tissues or the contaminated surfaces of medical and culinary devices and food products. Recently, the United States Food and Drug Administration approved phage cocktails against *Listeria monocytogenes* for use in ready to eat food as generally recognized as safe, further encouraging topical applications^33^.

Phage lysates generated by induction or propagation of temperate phage to the host strain harboring a plasmid or phagemid containing CRISPR/Cas system are mixtures of bacterial components including bacterial DNA, proteins, and cell wall components, as well as transducing phage particles. As demonstrated in Fig. 5, phage lysates generated from the strain RF122Δ*nuc* contained superantigens and cytolysins expressed from the chromosome of RF122. These aspects will clearly raise a regulatory compliance concern on pharmaceutical use of phage lysates containing CRISPR/Cas antimicrobials in Western clinical settings. To alleviate this concern, we generated RF122 containing multiple toxin gene deletions resulting in loss of 10 superantigen and 11 cytolysin genes (RF122-19Δ*nuc*) by using the modified pMAD-secY temperature sensitive shuttle vector system established in this study. Therefore, phage lysates generated from RF122-19Δ*nuc* did not show any harmful effects associated with superantigens and cytolysins. Furthermore, RF122-19Δ*nuc* can be used as a virulence factor-free host strain to propagate ϕSaBov-Cas9-nuc, without risk of spreading superantigen and cytolysin genes by temperate phage-mediated generalized transduction. One may still argue that the virulence factor-free host strain would not be an ultimate solution because phage lysates generated from RF122-19Δ*nuc* may still contain other uncharacterized virulence factors in host chromosomal segments. Our previous study showed that excision and packaging of host chromosomal segments by ϕSaBov was highly specific to MGEs within the strain RF122 background, and not in other *S. aureus* strain backgrounds such as RN4220 and MW2^23^. A recent study demonstrated that the integrase and terminase small subunit (TerS) encoded in *Staphylococcus aureus* pathogenicity islands (SaPI) induced sequence-specific excision of host chromosome unlinked to phage DNA which packaged into transducing phage particles by the terminase large subunit (TerL) encoded by the helper phage^34^. Genome sequence analysis of strain RF122 showed a single copy of the *terL* gene associated with the genome of ϕSaBov, and two copies of the *terS* genes, one with ϕSaBov and the other with SaPIbov1^32^. We are currently seeking to remove the redundant integrases and TerS gene to prevent excision of host chromosome mediating generalized transduction by ϕSaBov.

Phage absorption, an important process that determines the host specificity of phage, is mediated by the phage tail module. The phage tail module is typically composed of tape measurement protein, minor tail protein, baseplate protein, cell wall hydrolase, and tail fiber protein. The baseplate protein is linked with the tail fiber protein which can be extended out to search the host receptors such as lipopolysaccharides or the outer membrane porin protein C^25^,^26^. This contact triggers conformational changes in the baseplate protein of phages, causing irreversible binding of tail fibers to the outer core of lipopolysaccharides and penetration of inner tail tube to bacterial membrane allowing ejection of phage DNA^25^. A recent study in *Pseudomonas aeruginosa* Pap1 phage demonstrated that a single nucleotide mutation in phage tail fiber protein resulted in altered host specificity^27^. These results suggest that host specificity of phage could be modulated by altering the phage tail module. The tail fiber protein of ϕ11 (orf50) contains 370 amino acid residues in which the N-terminal part of amino acid residues from 1 to 120 are highly similar to the putative tail fiber protein of ϕSaBov (orf56) with 74% identity (85/120) and 85% positivity (102/120). Recent studies demonstrated that the baseplate protein of ϕ11 (Gp45) plays an essential role in phage absorption to *S. aureus* by mediating interaction with the N-acetyl-glucosamine (GlcNAc) in the peptidoglycan^35^,^36^. Interestingly, the baseplate protein of ϕSaBov (orf51) showed high similarity with that of ϕ11 (Gp45) with 92% identity (587/636) and 96% positivity (615/635). These suggest a possibility that the baseplate protein is linked with the N-terminal part of tail fiber protein and the C-terminal part of tail fiber protein interact with the specific receptor in the host. This could explain the partial improvement of host specificity of ϕSaBov by complementing the plasmid harboring the gene encoding tail fiber protein of ϕ11 (pTFϕ11) because the high amino acid sequence homology in the baseplate proteins and the N-terminal part of tail fiber proteins between ϕSaBov and ϕ11 allows the interchangeable linkage of tail fiber protein from both ϕSaBov and pTFϕ11 to the baseplate protein of ϕSaBov, but causes competitions which results in partial improvement of host specificity. These results suggest that elucidating the interaction between the baseplate protein and tail fiber protein may lead to generate a chimeric tail fiber protein that bridges the C-terminal receptor recognition domain of tail fiber protein to the baseplate protein of phage for developing recombinant phage delivery system for CRISPR/Cas9. Currently, we are pursuing either complete replacement of the *tif* gene within the ϕSaBov genome with that of a broad host range phages such as ϕ11, ϕ13, and ϕNM1 by allelic exchange or integration of CRISPR/Cas9 system into the genome of broad host range phage.

A phage therapy carrying CRISPR/Cas antimicrobials undoubtedly has great potential for alternative therapeutics, supplemental to conventional antibiotics, and prophylactic measurement against increasing antibiotic resistant pathogens. The genetic engineering strategy on both phage and host genome established in this study will be useful to create an efficacious and safe CRISPR/Cas9 antimicrobials platform broadly applicable to MRSA and other important pathogens.

## Methods

### Bacterial strains and growth conditions

All strains and plasmids used in this study are listed in Supplementa1 Table 1. *Staphylococcus aureus* strains were cultured in tryptic soy broth (TSB) or agar (TSA) plates (Difco) supplemented with chloramphenicol (10 μg/mL, Sigma-Aldrich) as necessary. *Escherichia coli* were grown in Luria-Bertani (LB) broth and agar plates supplemented with ampicillin (100 μg/mL, Sigma-Aldrich) as necessary.

### Plasmid construction

All oligos used in this study are listed in Supplemental Table 2. Synthetic oligos (CRISPR_f/CRISPR_r) containing promoter, pre-crRNA, and direct repeats flanked with *Bbs*I sites (CRISPR array) were annealed, ligated into pMK4 digested with *BamH*I and *EcoR*I, resulting pKS1. Synthetic oligos (spacer-nucf/spacer-nucr) containing a spacer sequence specific to the *nuc* gene followed by protospacer-adjacent motif (NGG) were annealed and ligated into pKS1 digested with *Bbs*I, resulting pKS2. The tracrRNA and the cas9 genes were amplified from the genomic DNA of *Streptococcus pyogenes* SF370 (ATCC) using oligos (tracrrnaf/cas9r), followed by digestion with *Afl*II and *Eag*I, and ligation into corresponding sites in modified pMAD-secY temperature sensitive shuttle vector, resulting pKS3. To program tracrRNA and the cas9 gene specific to *S. aureus,* CRISPR array specific to the *nuc* gene was amplified from pKS2 using oligos (leaderf/drr), digested with *Eag*I and *Sbf*I, and cloned into corresponding sites in pKS3, resulting pKS4.

### Allelic exchange construct

Integration of CRISPR-Cas9 system into the genome of ϕSaBov and marker-less deletion mutants of the *nuc* gene and 21 virulence genes were generated by allelic exchange using modified pMAD-secY temperature sensitive shuttle vector system established in this study by introducing a new multi-cloning site, a GFPuv reporter gene, a chloramphenicol resistant gene (*cat*), and an anti-sense *secY* gene controlled by a tetracycline inducible promoter into the pMAD system^37^ to improve screening process of allelic exchange (Supplemental Figure S1). Briefly, upstream and downstream fragments of target gene were amplified and cloned in modified pMAD-secY system in *E. coli*, followed by electroporation into *S. aureus* strains. The first homologous recombination was induced by culturing at 43 °C (non-permissive temperature for the replication of pMAD-secY), followed by culturing at 37 °C to promote the second recombination, resulting in allelic exchange^38^. The mutant candidates were screened by growth in TSA plate supplemented with anhydrous tetracycline (0.5 μg/ml), loss of GFP expression, and no growth in TSA plate supplemented with chloramphenicol, indicating the second recombination.

### Phage lysates

Phages were induced from the mid-exponential culture of strains by adding mitomycin C (1 μg/mL, Sigma-Aldrich) which induced clear lysis typically in 3 hours incubation at 30 °C with 80 rpm. The lysates were sterilized with syringe filers (0.22 μm, Nalgene). Phage lysates were generated by propagating phage to the mid-exponential culture of the same strains from which phages were initially induced, followed by filter sterilization of lysates. The number of transducing phage particles (TP) was determined by calculating the plaque-forming unit using soft agar (0.5%) overlay method or quantitative real time PCR. Briefly, phage lysates were treated with excessive Dnase I (Sigma-Aldrich) to remove chromosomal DNA contamination, followed by DNA extraction from phage particles using DNeasy kit (Qiagen) as described previously^23^,^24^. Quantitative real time PCR reaction was performed using SYBR green I master mix (Applied Biosystems), primer sets specific to phages, and a serial dilution of phage DNA templates. The absolute copy number of phage DNA was calculated by interpolation of the threshold cycle from phage DNA template to the standard curves generated from cloned plasmid templates.

### *In vitro* efficacy tests

The phage stock generated from the mitomycin C induction was propagated to the RF122-19Δ*nuc*ϕSaBov-Cas9-nuc strain for 5 times to remove the mitomycin C contamination in the phage lysates. The mid exponential culture of recipient strains was harvested by centrifugation and adjusted to 1 × 10^6^ CFU/mL in PBS. A test tube killing assay was performed in 1 mL of reaction mixtures consisting of 100 μL of recipient cell suspension, 20 μL of serially diluted phage lysates, and 880 μL TSB, and incubated at 37 °C. The number of viable cells at each time point was determined by serial dilution and plating onto TSA plates. For *in vitro* killing under nutritionally limited conditions, an empty antibiotic disc was placed in sterile petri dish and inoculated with 100 μL of recipient cell suspension in PBS (1 × 10^5^ CFU), followed by 20 μL of serially diluted phage lysates. After an 8 hr incubation at 37 °C, the viable cells were recovered by blotting the disk onto TSA plates.

### *In vivo* efficacy tests

All animal experiments were performed in compliance with a protocol reviewed and approved by the Institutional Animal Care and Use Committee at the Mississippi State University (14–040). The back of C57BL/6 mice (6 to 8 week old, female, Harlan laboratory) were shaved with electric razor, depilated with Nair cream, and decontaminated with 70% ethanol swab. For intradermal infection, 100 μl of bacterial suspension in PBS containing 1 × 10^5^ CFU was intradermally injected to the shaved skin. After 6 h, 100 μl of phage stock containing 5 × 10^7^ transducing phage particles was intradermally injected to the infected skin. For skin surface infection, shaved skin was topically infected with 5 μL of bacterial suspension containing 2 × 10^4^ CFU. After 1 h, phage stock containing 1 × 10^6^ transducing phage particles/μl was applied to the infected skin in solution (10 μl) or mixing with a hydrogel (Dynarex). After 24 h, mice were euthanized with CO2, and infected skin was excised and homogenized using Omni TH tissue homogenizer (OMNI international). Homogenates were serially diluted and plated on to BHI plate to determine the number of viable cells.

### Phage spot and absorption assay

For phage spot assay, recipient strains grown to the mid-log phage in BHI were harvested, adjusted to 1 × 10^6^ CFU/ml, mixed with soft agar (0.5% w/v), and overlaid onto the BHI plate. Ten microliters of phage lysates (1 × 10^8^ pfu/ml) was dropped onto the lawn culture.

For phage absorption assay, 100 μL of the mid-exponential phase recipient *S. aureus* (5 × 10^8^ CFU/ml) was mixed with 100 μL of phage lysates (2.5 × 10^6^ PFU/ml) in phage buffer (50 mM Tris-HCl pH 7.5, 100 mM NaCl, 10 mM CaCl2) and incubated for 15 min at 37 °C. The phages bound to the bacterial cells was removed by centrifugation at 13,000 rpm for 15 min. The PFU of the supernatant containing the unbounded phage was determined by the soft agar (0.5%) overlay method. The phage absorption (% input) was calculated by [1 − (the number PFU of unbound phage/1 × 10^5^ input PFU)] × 100. Each absorption assay was repeated at least three times.

### Toxin detection in phage lysates

Heparinized human venous blood was collected from healthy volunteers. All methods used in this study were carried out in accordance with the approved guidelines and all experimental protocols were approved by the Institutional Review Board for Human Subjects at the Mississippi State University (12–041). Informed written consent was obtained from all volunteers. Peripheral blood mononuclear cells (PBMCs) were isolated by density gradient centrifugation using Ficoll-Histopaque (Sigma-Aldrich). Purified PBMCs were adjusted to 2 × 10^6^ cell per well in 96 well cell culture plate in RPMI1640 medium supplemented with 10% FBS. Phage lysates prepared from RF122Δ*nuc* or RF122-19Δ*nuc* was added to the wells. To detect superantigens in phage lysates, proliferation of T cell was measured using a [^3^H]-thymidine incorporation assay as described previously^39^. To detect cytotoxins in phage lysates, cytotoxicity of cells were measured using propidium iodide incorporation assay using LIVE/DEAD Cell-mediated Cytotoxicity kit (ThermoFisher).

## Additional Information

**How to cite this article:** Park, J. Y. *et al*. Genetic engineering of a temperate phage-based delivery system for CRISPR/Cas9 antimicrobials against *Staphylococcus aureus. Sci. Rep.* **7**, 44929; doi: 10.1038/srep44929 (2017).

**Publisher's note:** Springer Nature remains neutral with regard to jurisdictional claims in published maps and institutional affiliations.

## Supplementary Material

Supporting Information
