# Supplementary material for: Genetic engineering of a temperate phage-based delivery system for CRISPR/Cas9 antimicrobials against Staphylococcus aureus
Source: Sci Rep. 2017 Mar 21;7:44929. doi: 10.1038/srep44929 (PMC5359561; doi:10.1038/srep44929)
Supplement: Supporting Information [file srep44929-s1.pdf]

Genetic engineering of a temperature phage-based delivery system for CRISPR/Cas9 antimicrobials against *Staphylococcus aureus*

Bo Youn Moon[1,2$], Joo Youn Park[1$], Juw Won Park[3], Justin A. Thornton[4], Yong Ho Park[2], Keun Seok Seo[1*]

[1]Department of Basic Sciences, College of Veterinary Medicine, Mississippi State University, Mississippi State, MS 39762, United States

[2]Department of Microbiology, College of Veterinary Medicine and BK21 Program for Veterinary Science, Seoul National University, Seoul 151-742, South Korea

[3] Department of Computer Engineering and Computer Science, University of Louisville, KY 40208, United States

[4]Department of Biological Sciences, Mississippi State University, Mississippi State, MS 39762, United States

[$]Both authors equally contributed to the work.

*Corresponding author

Correspondence and requests for materials should be addressed to Keun Seok Seo

e-mail) seo@cvm.msstate.edu

Tel) 1-662-325-1018

Fax) 1-662-325-1031

Supplemental Table 1. Bacterial strains and plasmids used in this study

Strain	Description	Reference or source
Staphylococcus aureus		
RF122	Bovine isolate, CC151, Lysogenized with ϕSaBov	[1]
RF122Δ*nuc*	The *nuc* gene deletion mutant of RF122	This study
RF122Δ*nuc* ϕSaBov-Cas9-nuc	Integration of CRISPR-Cas9 system specific to the *nuc* gene into the genome of ϕSaBov lysogenized in RF122	This study
RF122Δ*nuc* ϕSaBov-Cas9-null	Integration of CRISPR-Cas9 system without spacer sequence into the genome of ϕSaBov lysogenized in RF122	This study
RF122-19	10 cytotoxins and 11 superantigen gene deletions mutant of RF122	This study
RF122-19Δ*nuc*	The *nuc* gene deletion mutant of RF122-19	This study
RF122-19Δ*nuc*ϕSaBov-Cas9-nuc	Integration of CRISPR-Cas9 system specific to the *nuc* gene into the genome of ϕSaBov lysogenized in RF122-19	This study
RF122-19Δ*nuc*ϕSaBov-Cas9-null	Integration of CRISPR-Cas9 system without spacer sequence into the genome of ϕSaBov lysogenized in RF122-19	This study
RF122-19Δ*nuc*ϕSaBov-pTF11	Complementation of ϕ11 tail fiber protein gene in RF122-19Δ*nuc*	This study
RF122-19Δ*nuc*ϕSaBov-Cas9-nuc-pTF11	Complementation of ϕ11tail fiber protein gene in RF122-19Δ*nuc*ϕSaBov-Cas9-nuc	This study
CTH96	Bovine isolate, CC151, susceptible to ϕSaBov	[2]
CTH96Δ*nuc*	The *nuc* gene deletion mutant of CTH96	This study
CTH96pGFP	Expression of green fluorescence protein on CTH96	This study
NRS382	Human MRSA USA100, ST5	[3]
MN PE	Human MRSA USA200, ST36	[4]
DAR1809	Human MRSA USA300, ST8	[4]
MW2	Human MRSA USA400, ST1	[5]
Escherichia coli		
DH5α	Cloning host of pMAD and pMK4	Life Technologies
Top10	Cloning host of pCR4	Life Technologies
**Plasmid**	**Description**	**Reference**
Modified pMAD-secY	Temperature sensitive shuttle vector system	This study

pCR4-TOPO	TA cloning vector	Life Technologies
pMK4	High copy number vector for complementary	[6]
pKS1	Cloning of synthetic oligos containing a promoter, pre-crRNA, and two BbsI restriction sites flanked with a direct repeat (CRISPR array) into pMK4	This study
pKS2	Cloning of the spacer sequence specific to the *nuc* gene into pKS1	This study
pKS3	Cloning of a tracrRNA and Cas9 into modified pMAD-secY	This study
pKS4	Cloning of PCR product containing CRISPR array with spacer sequence specific to the *nuc* gene amplified from pKS2 into pKS3	This study
pKS5	Cloning of SAB1737 and SAB1738 into pKS4	This study

Supplemental Table 2. Oligonucleotides used in this study

Name	Sequences (5' to 3')
Cloning of Cloning of CRISPR array containing a promoter, pre-crRNA, DR, and BbsI sites	
CRISPR_f	GATCCCGGCCGACGTGAACTATATGATTTTCCGCAGTATA TTTTAGATGAAGATTATTTCTTAATAACTAAAAATATGGT ATAATACTCTTAATAAATGCAGTAATACAGGGGCTTTTCA AGACTGAAGTCTAGCTGAGACAAATAGTGCGATTACGAA ATTTTTTAGACAAAAATAGTCTACGAGGTTTTAGAGCTAT GCTGTTTTGAATGGTCCCAAAACGGGTCTTCGATCGATCG ATCGAAGACGTTTTAGAGCTATGCTGTTTTGAATGGTCCC AAAACCTTGCAGGGG
CRISPR_r	AAT TCC CCT GCA AGG TTT TGG GAC CAT TCA AAA CAG CAT AGC TCT AAA ACG TCT TCG ATC GAT CGA TCG AAG ACC CGT TTT GGG ACC ATT CAA AAC AGC ATA GCT CTA AAA CCT CGT AGA CTA TTT TTG TCT AAA AAA TTT CGT AAT CGC ACT ATT TGT CTC AGC TAG ACT TCA GTC TTG AAA AGC CCC TGT ATT ACT GCA TTT ATT AAG AGT ATT ATA CCA TAT TTT TAG TTA TTA AGA AAT AAT CTT CAT CTA AAA TAT ACT GCG GAA AAT CAT ATA GTT CAC GTC GGC CGG
Cloning of spacer sequencing into pKS1	
spacer-nucf	AAACTGCAAAGAAAATTGAAGTCGAGTTTGACAAGT
spacer-nucr	TAAAACTTGTCAAACTCGACTTCAATTTTCTTTGCA
spacer-esxAf	AAACTGCACAAGGTGAAATTGCAGCGAACTGGGAGT
spacer-esxAr	TAAAACTCCCAGTTCGCTGCAATTTCACCTTGTGCA
Cloning of trac-RNA and Cas9 into modified pMAD-secY	
tracrrnaf	GATCCTTAAGTGATCCCTTGAAAGATTCTGT
cas9r	GATCCGGCCGTCAGTCACCTCCTAGCTGACTCA
Cloning of CRISPR array with a spacer sequence specific to the *nuc* into pKS3	
leaderf	GCAGGTCGACGGATCCCGGCCGACGTGAACTATATGATTT TCCGC
drr	AACGACGGCCAGTGAATTCCCCTGCAGGGTTTTGGGACCA TTCAAAACAGCATAGCTCTAAAACGTCTTCGATCGATCGA TCGAAGACCC
Cloning of SAB1737 and SAB1738 into pKS4	
1737upf	GATCGTCGACTTATGCTTCACTCCATTTC
1737upr	GATCCTTAAGATGGGCAGTGTTGTAATTAT
1738dnf	GATCCCTGCAGGTGTTGTTGCATTAAATCACT
1738dnr	GCGCAGATCTTGATATTTAGAGGTGGCACA
Generating the *nuc* gene deletion mutant	
nucupf	GATCGTCGACGTTAACACTTTAAGCAAACCGCATC
nucupr	GATCACGCGTAACTAACACCTCTTTCTTTTTAG
nucdnf	GATCGAATTCTGCTCATTGTAAAAGTGTCACTGCT

nucdnr	GATCCCCGGGATACGTCGCTACCATCTTCT
Generating the *esxA* gene deletion mutant	
esxAupf	GATCGTCGACTGTGATAAGAGGCTGATC
esxAupr	GATCACGCGTCCCGTAAGATTGCGATTTTG
esxAdnf	GATCGAATTCGCAATTCCAACAACTTAGTCC
esxAdnr	GATCCCCGGGGATTTATTGTATGTTTGGAACC
Generating α-hemolysin (*hla*) deletion mutant	
hlaupf	GCGCGGATCCTTACCTCATATAGTGTCATG
hlaupr	GCGCGTCGACGAAAGGTACCATTGCTGGTC
hladnf	GCGCGAATTCGTCAATTTAGAATATTGCAG
hladnr	GCGCAGATCTAATGCCTCTAACTAAAAACC
Generating β-hemolysin, leukocidin G/H deletion mutant	
1874f	GCGCGGATCCCTTAATTCCGATTACATTTG
1874r	GCGCGTCGACGTGCCTTTATTAACATTAAG
1876f	GCGCGAATTCCTTCAAGTCATTCGCAATAA
1876r	GCGCAGATCTGTATCAACGATCTTATTAAC
Generating gamma hemolysin ACB deletion mutant	
hlgaupf	TAATGGATCCACCGTTGATTCTCAATCG
hlgaupr	TGAAGTCGACCATCTTAACAACTAGGGC
hlgbdnf	GCGCGAATTCGGCTTTGTGAAACCTAATCC
hlgbdnr	GCGCAGATCTGGTCGTCACAATTACTGTTG
Generating leukocidin D/E (*lukDE*) deletion mutant	
lukdeupf	GCGCGGATCCCGAATTTGGAGATGGCTGC
lukdeupr	GCGCGTCGACCTAATCCTGGAGTATAACTG
lukdednf	TGATGAATTCCTATTGCCCGTTAAACGG
lukdenr	ATTGAGATCTCCTGTCGGTTTACTCATTG
Generating leukocidin M/F\` (*lukMF\`*) deletion mutant	
lukmfupf	GCGCGGATCCTTCGTATAGGCTTTATAG
lukmfupr	GCGCGTCGACCTCCAATGTTATATCCTA
lukmfdnf	GCGCGAATTCCTACTTCCTAGATACCGT
lukmfdnr	GCGCAGATCTGAATAGCTTAACAACGTA
Generating Enterotoxin gene cluster (*egc*) deletion mutant	
egcupf	TCTTGATACGTATTTGACACTTGC
egcupr	AGCTATACGAGTTTGATGGTTCTG
egcdnf	CAGAACCATCAAACTCGTATAGCTAACTAAGCGACTCAG ATAATAGAC
egcdnr	AGAGTTGTTACAGTCGCTACACC
Generating staphylococcal enterotoxin C deletion mutant	

secupf	ATGAATTC CTGTGGATTTAGAAATAAGG
secupr	CCAACATTCCCAAGAAGTATC
secdnf	GATACTTCTTCTTGGGAATGTTGGAAGAATGGATAATGTT AATCC
secdnr	TTATCCATGGCAAGCATCAAAC
Generating staphylococcal enterotoxin like toxin L deletion mutant	
selupf	GATATATTTGAAAGGTAAGTACTTCG
selupr	AGTGTAGTATTCCATATGAATGATGGT
seldnf	ACCATCATTCATATGGAATACTACACTATACAAAAGGTTA TAGGAAGAGTT
seldnr	CAATTTCTACAGATATGACTCCC
dself	GTCATGTTTCGGTTGATAGG
dselr	TGTACAAATGGACTTAAGATATAGCG
Generating toxic shock syndrome toxin deletion mutant	
tstupf	GCGCGGATCC ACTACATGTACTTACCAATGCG
tstupr	GCGCGTCGACGCAGAAATTAATTAATTTACCACTTTTTCT
tstdnf	GCGCGAATTCCAAAGGGCTTACGATGAAAAATTTCAT
tstdnr	GCGCAGATCTAACCAATTACCAAATTCTCCATGC
Generating staphylococcal enterotoxin like toxin X deletion mutant	
selxupf	TGTCGATGCTATGGATAGTGAGG
selxupr	TAATTACCTCCTTGATGTAAAGC
selxdnf	GCTTTACATCAAGGAGGTAATTATATCGCTAATACTTTGA AAGTTAGG
selxdnr	TCAAATGTAGCAGTATACATTAATTGCG
Complementation of the tail fiber protein of ϕ11	
synf	GCTACTGCAGATCCCATTATGCTTTGGCAG
synr	GGGTTTCACTCTCCTTCTACA
ϕ11tailf	AGGAGAGTGAAACCCATGTACAAAATAAAAGATGTTGAA AC
ϕ11autor	GCTAGGATCCCTAACTGATTTCTCCCCATAAG
Confirmation of deleted cytotoxins and superantigens	
lukdf	TTGCCAGTCAACTTCATAAGTAGATGT
lukdr	GCGCGTGGTAACTTTAACCC
lukef	TTTTTACCATCAGGCGTAACA
luker	ACGAATGATTTGGCCATTCC
lukmf	TGGAGGTAAATTCCAGTCAGCA
lukmr	TGTCGCGATAAAAGACGGATT
lukf′f	ACTGGTGGATTGAACGGGTC
lukf′r	ATCCAGTGCAAGTTGTTCCAAA

lukgf	GACTTTGCACCAAAAAATCAGGAT
lukgr	AGGTGCATAATGTATTCCAGGTCT
lukhf	GCGTCATCATTATCATGTGCAA
lukhr	CAAGGCTCAATTCATTCAAATT
hlgaf	CCAATCAGCGCCATCAATC
hlgar	CCAGTTGGGTCTTGTGCAAAT
hlgbf	CGTTGCTACTTCTATGGCAT
hlgbr	ACATTGTATTTAGCTCCCCAA
hlgcf	ATGTTAAAGCTATGCGATGGCC
hlgcr	AAGAGGTGGTAACTCACTGTCTGGA
hlaf	TGTTTGTTGTTTGGATGCTTTTCT
hlar	GGTTTAGCCTGGCCTTCAGC
hlbf	CACCTGTACTCGGCCGTTCT
hlbr	TATACATCCCATGGCTTAGGTTTTTC
segf	GGTAACAATCGACAATAGACAATCACTT
segr	TCCAGATTCAAATGCAGAACCAT
senf	TGGACTGTATTTTGGAAATAAATGTGT
senr	GCTCCCACTGAACCTTTTACGT
seuf	AGCGAGTGAATTCTCTGGTTTAATG
seur	TTGTGCTGTTATGTTTTTCATATTGG
seif	TGGCATTGATTATAATGGTCCTTG
seir	GCCTTTACCAGTGTTATTATGACC
semf	TCAGTTTCGACAGTTTTGTTGT
semr	CAGCTCAAGAAATTGATACTAAATTAAGAAG
seof	ACTACAGATAAAAAGAAAGTTACTGCAC
seor	CATCAATATGATAGTCTGATGAATCTATTG
secf	CAACCAGACCCTACGCCAGA
secr	TGTTATAAATTAAATCATGTGCCAAAAA
self	TAATTATCAATGGCAAGCATCAAAC
selr	CACTCCCCTTATCAAAACCGC
tstf	TGAATTTTTTTATCGTAAGCCCTTTG
tstr	GGAAATGGATATAAGTTCCTTCGCT
selxf	TCAACACAAAATTCCTCAAGTGT
selxr	GCGACTCTAATGTATATTTACCGCC
qRT-PCR	
qintf	CATCACTGGTGGACGCTTTG
qintr	AATGCATCGAGCGCTTTTTC
qcas9f	CGGAAGCGACTCGTCTCAA

qcas9r	CAAATACGATTCTTCCGACGTGTAT
qϕ11intf	TCTTTCTGTTGACTATGCACGATCT
qϕ11intr	TTTTGGCGTAATTGATAACTGCTT
qϕnm1intf	TTCTGTTGGCTATGCACGATCT
qϕnm1intr	TTTTGGCGTAATTGATAACTGCTT

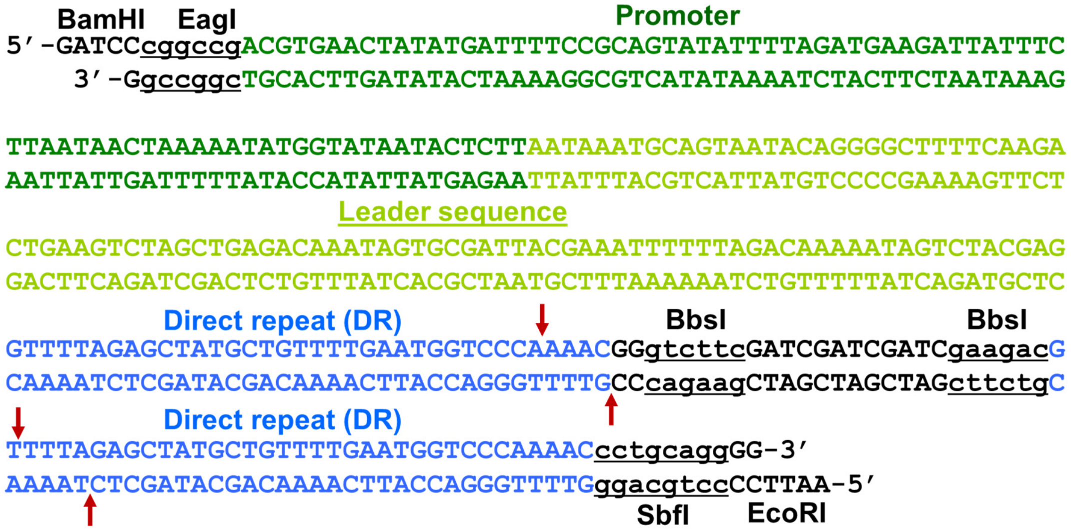

Supplemental Figure S1. Synthetic oligos containing the promoter, leader, and direct repeat (DR) sequence cloned in *BamH*I/*EcoR*I sites in the pMK4 plasmid to generate pKS1. The color scheme is matched in the Figure 1. The arrow indicates the cleavage site by the BbsI restriction enzyme.

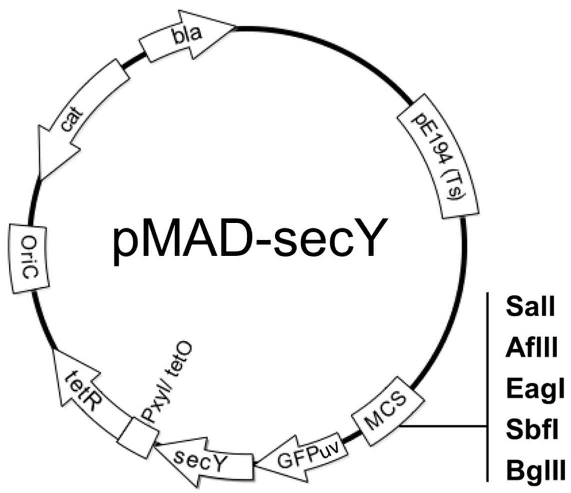

Supplemental Figure S2. A schematic map of modified pMAD-secY system. For an efficient screening process of double-crossover event, genes encoding chloramphenicol resistance (*cat*), anti-sense-secY controlled by teteracycline inducible promoter (*secY*), and green fluorescent protein UV variant (GFUuv) were added to the original pMAD system.

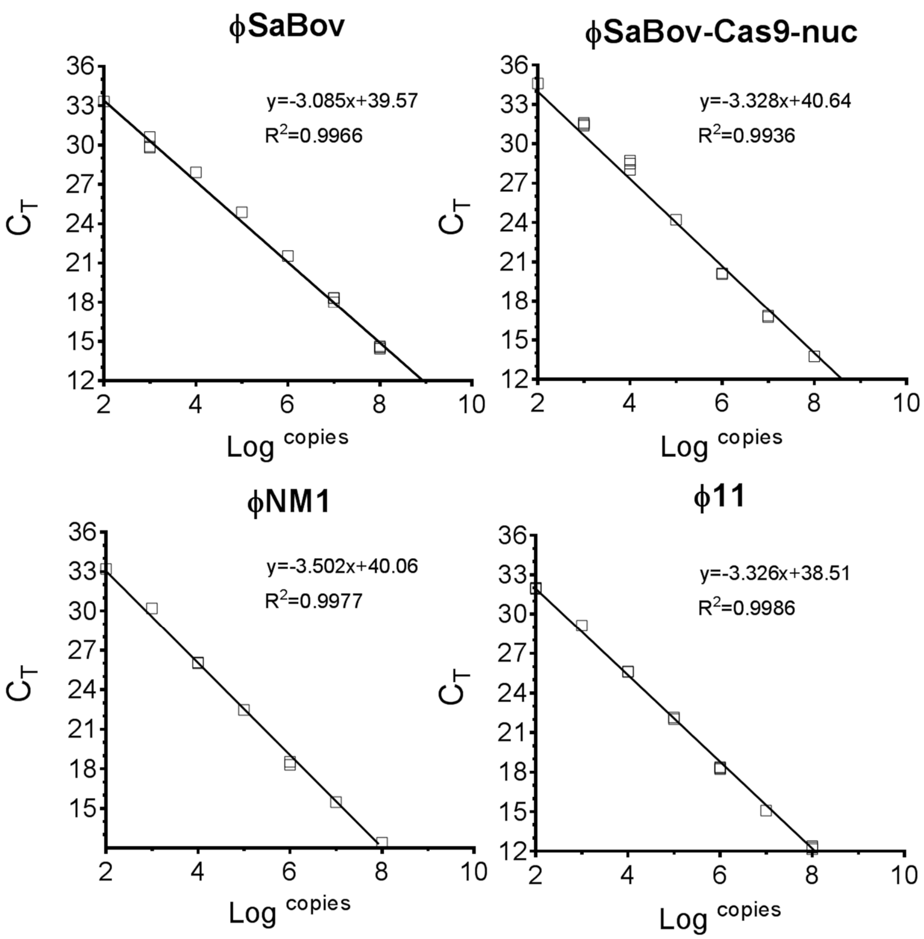

Supplemental Figure S3. Standard curves to determine the absolute copy number of phage DNA. The integrase gene for each phage was amplified and cloned into pCR4-TOPO (Life technologies). A serial dilution of cloned plasmid was used in quantitative real time PCR. Standard curves were generated by linear regression analysis to calculate the slope, intercept, and correlation coefficient (R^2) using Microcal OriginPro (Microcal origin, Version 7.5).

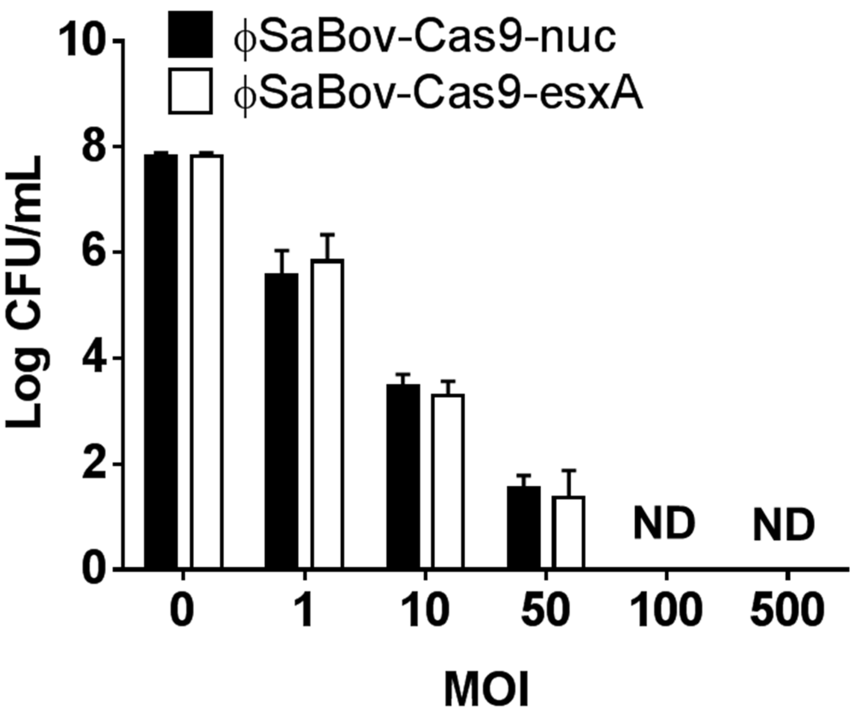

Supplemental Figure S4. The efficacy of ϕSaBov-Cas9 system targeting the *esxA* gene (ϕSaBov-Cas9-esxA). A mid exponential culture of *S. aureus* strain CTH96 (1×10^5 CFU) was treated at various MOIs of ϕSaBov-Cas9-nuc or ϕSaBov-Cas9-esxA for 6h. Viable cells were recovered on BHI plates. Data point represent the average of triple measurements which were repeated in three independent experiments. The killing efficacy of ϕSaBov-Cas9-esxA was not significantly different from ϕSaBov-Cas9-nuc in the student t-test. ND indicates no viable cell was detected.

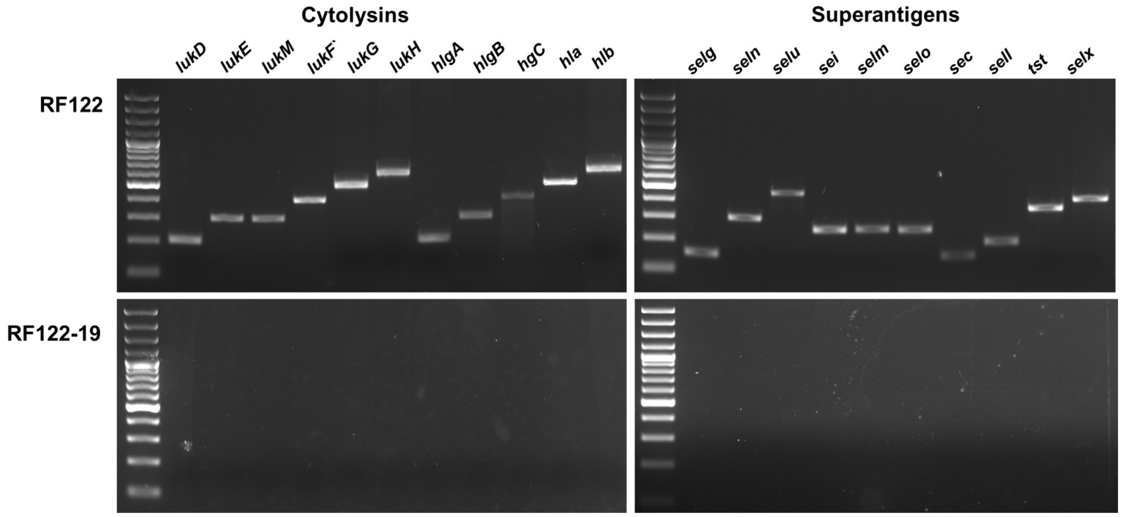

Supplemental Figure S5. PCR analysis of cytolysins and superantigens genes. The strain RF122 harbors 11 cytolysins (*hla, hlb, hlgA, hlgB, hlgC, lukD, lukE, lukG, lukH, lukM, lukF'*) and 10 superantigens (*sec, seg, sei, selm, seln, selo, selu, sell, tst1, selx*) gene in the chromosome (RF122 panel). The 11 cytolysin and 10 superantigen genes were removed from RF122 chromosome without antibiotic selection markers by allelic exchange using modified pMAD-secY system which was confirmed by PCR analysis (RF122-19 panel).

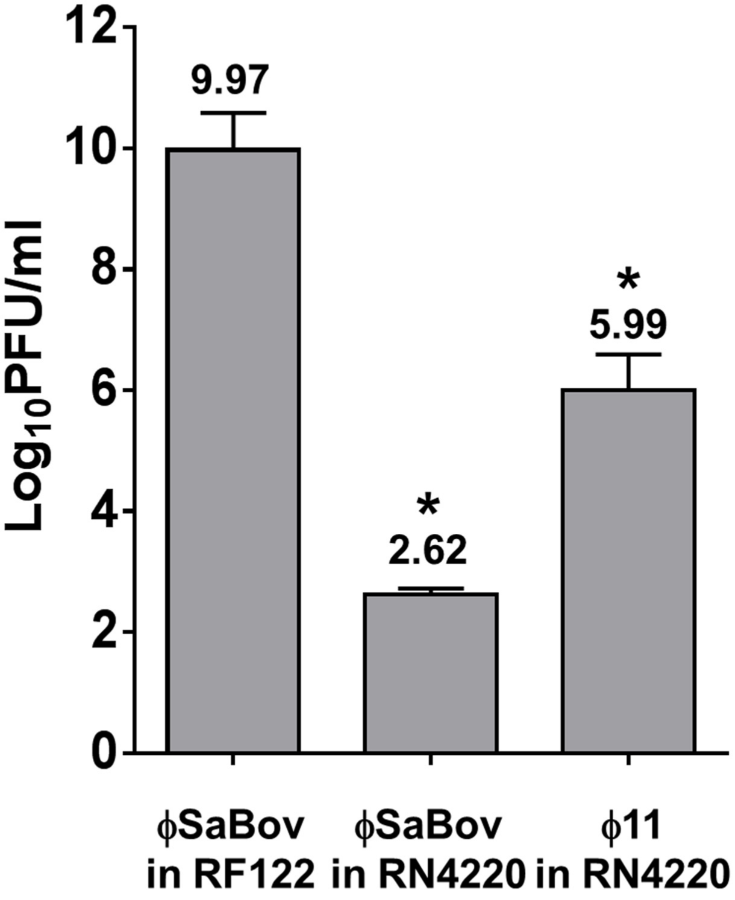

Supplemental Figure S6. The ϕSaBov lysogenized in the RF122 and RN4220 strains were induced and the number of transducing phage particles of in the phage lysates was determined by calculating the plaque forming unit (PFU) using semi-solid agar overlay method. The bar graph indicates the average and SEM combined from triple measurements of three independent experiments (n=9). Asterisk indicates statistical significance in student *t*-test, compared to the results from ϕ11 ($p<0.001$).

Reference

1 Herron-Olson, L., Fitzgerald, J. R., Musser, J. M. & Kapur, V. Molecular correlates of host specialization in *Staphylococcus aureus*. *PloS one* **2**, e1120, doi:10.1371/journal.pone.0001120 (2007).

2 Joo, Y. S., Fox, L. K., Davis, W. C., Bohach, G. A. & Park, Y. H. *Staphylococcus aureus* associated with mammary glands of cows: genotyping to distinguish different strains among herds. *Veterinary microbiology* **80**, 131-138 (2001).

3 McDougal, L. K. *et al.* Pulsed-field gel electrophoresis typing of oxacillin-resistant *Staphylococcus aureus* isolates from the United States: establishing a national database. *Journal of clinical microbiology* **41**, 5113-5120 (2003).

4 Moon, B. Y. *et al.* Phage-mediated horizontal transfer of a *Staphylococcus aureus* virulence-associated genomic island. *Scientific reports* **5**, 9784, doi:10.1038/srep09784 (2015).

5 Baba, T. *et al.* Genome and virulence determinants of high virulence community-acquired MRSA. *Lancet (London, England)* **359**, 1819-1827 (2002).

6 Sullivan, M. A., Yasbin, R. E. & Young, F. E. New shuttle vectors for *Bacillus subtilis* and *Escherichia coli* which allow rapid detection of inserted fragments. *Gene* **29**, 21-26 (1984).
